# Recent Changes in Trends of Nationwide Incidence of Glaucoma and Associated Visual Impairment in South Korea

**DOI:** 10.3390/jcm14165691

**Published:** 2025-08-12

**Authors:** Sooyeon Choe, Chen Xi, Joonhyung Kim, Ahnul Ha, Young Kook Kim

**Affiliations:** 1 Department of Ophthalmology, Chungnam National University College of Medicine, Daejeon 35015, Republic of Korea; 2 Department of Ophthalmology, Chungnam National University Hospital, Daejeon 35015, Republic of Korea; 3 Ranelagh Center for Biosocial Informatics, Seoul National University College of Medicine, Seoul 07061, Republic of Korea; 4 Department of Ophthalmology, CHA Bundang Medical Center, CHA University School of Medicine, Sungnam 13497, Republic of Korea; 5 Department of Ophthalmology, Jeju National University College of Medicine, Jeju 63241, Republic of Korea; 6 Department of Ophthalmology, Jeju National University Hospital, Jeju 63241, Republic of Korea; 7 Department of Ophthalmology, Seoul National University Hospital, Seoul 03080, Republic of Korea; 8 Department of Ophthalmology, Seoul National University College of Medicine, Seoul 03080, Republic of Korea

**Keywords:** glaucoma, visual impairment, incidence trends, epidemiology

## Abstract

**Background/Objectives:** We analyzed recent changes in the incidence of glaucoma and associated visual impairment (VI) in Korea over a 16-year period. **Methods:** We utilized nationwide, longitudinal cohort data from the Korea National Health Insurance System (KNHIS) National Health Screening Cohort from 2004 to 2019 to evaluate the age-standardized incidence rate (SIR) of glaucoma and glaucoma-related VI. VI was determined based on KNHIS health examinations and the National Registry for Visual Disability. The incidence rates were estimated per 100,000 person-years. Joinpoint regression analysis was applied to assess significant changes in incidence trends, and subgroup analyses were conducted by age and sex. **Results:** The age-SIR of glaucoma increased from 864.1 per 100,000 in 2004 to a peak of 1101.1 in 2016, followed by a decline to 978.9 in 2019. Joinpoint regression identified a significant rise from 2007 to 2015 (annual percent change [APC]: +3.92%), with a subsequent decline (APC: –3.30%). The incidence of glaucoma-related severe VI decreased from 26.23 per 100,000 in 2004 to 8.76 in 2012, remaining stable thereafter at 12.49 in 2019. The VI-to-incidence ratio also declined from 0.030 in 2004 to 0.009 in 2012, which remained stable thereafter at 0.013 in 2019. Females consistently exhibited higher glaucoma incidence but lower VI rates than males. **Conclusions:** These recent trends highlight the evolving landscape of glaucoma epidemiology in Korea and underscore the need for sustained early detection efforts and optimized patient management.

## 1. Introduction

Glaucoma is a progressive optic neuropathy and one of the leading causes of irreversible blindness worldwide [1,2]. The number of glaucoma patients is projected to exceed 110 million by 2040 due to aging populations and demographic transitions [3]. In South Korea, the incidence of glaucoma nearly doubled between 2002 and 2013 [4], and the number of patients diagnosed with glaucoma increased by more than 120% between 2010 and 2019 [5]. This increase in incidence may be attributed to the rapid aging of the Korean population, improved accessibility to ophthalmic care, and the widespread use of advanced diagnostic technologies, such as optical coherence tomography (OCT).

The increasing prevalence of diagnosed glaucoma may be paralleled by a rising burden of glaucoma-related visual impairment (VI). A previous study by Sun et al. reported a relative reduction in the prevalence of blindness due to glaucoma, especially in high-income countries [6]. However, there remains limited evidence on whether this trend is reflected in national-level data, particularly in Asian countries where normal-tension glaucoma predominates. To date, there is a lack of comprehensive population-based analyses on the visual disability burden among patients newly diagnosed with glaucoma in Korea.

In this context, we conducted a nationwide, longitudinal retrospective cohort study using the Korean National Health Insurance Service (KNHIS) database to assess the trends in the incidence of glaucoma and its associated severe VI over a 16-year period [7,8]. By analyzing age-standardized data and applying joinpoint regression and spline modeling, we aimed to identify changes in temporal patterns and provide updated epidemiologic evidence on the burden of glaucoma in Korea.

## 2. Methods

### 2.1. Data Design and Data Source

This study utilized a 16-year database from the KNHIS from 2004 to 2019, which provides comprehensive health coverage to over 97% of the Korean population. The KNHIS database encompasses a wide range of healthcare information, including patient demographics, medical diagnoses, treatment records, and healthcare utilization data [7,8]. In this study, we identified individuals newly diagnosed with glaucoma by extracting the medical records of patients who had been assigned an International Classification of Diseases, 10th Revision (ICD-10) diagnosis code of H40, H42, H44.5, or Q15.0 and had received treatment with antiglaucoma medications or had undergone glaucoma surgery. Additionally, cases of glaucoma-related VI were identified using the ICD-10 code H54.

### 2.2. Participants

The study population comprised individuals aged 40 years and older who received a new diagnosis of glaucoma between 1 January 2004 and 31 December 2019. To ensure the accuracy of incident case identification and to rule out other possible causes of sight-threatening diseases, we excluded any individuals with a recorded diagnosis of glaucoma, diabetic retinopathy, and/or age-related macular degeneration between 2002 and 2003. Also, those with a prior history of severe VI were excluded to assess glaucoma-attributed severe VI.

### 2.3. Outcome

The primary outcome measure was the annual incidence rate of glaucoma, calculated per 100,000 individuals in the population. The secondary outcome focused on trends in glaucoma-related severe VI over the study period. Severe VI was identified using data from the KNHIS health checkup records and the National Disability Registration System [9]. The criteria included a corrected visual acuity worse than 6/60 in the better-seeing eye, as recorded during health screenings. Additionally, legal severe VI was defined as a best-corrected visual acuity of 6/100 or worse in the better eye, or a visual field restricted to 5 degrees or less from fixation in both eyes, persisting for at least six months despite appropriate treatment. Documentation by an ophthalmologist is required for certification of legal severe VI.

### 2.4. Follow-Up

Participants were observed between 1 January 2004 and 31 December 2019. People migrating out of the country or who had died were censored on the relevant date.

### 2.5. Statistical Analysis

Crude incidence rates were first calculated for each calendar year from 2004 to 2019 using mid-year population estimates derived from the Korean Resident Registration Data (http://kosis.kr; accessed on 1 July 2019). Incidence rates were reported per 100,000 person-years throughout this study. Female-to-male incidence ratios were also computed across all predefined age groups.

Temporal trends in glaucoma incidence and glaucoma-related VI were assessed using joinpoint regression analysis. This statistical method identifies time points at which significant changes in the slope of the trend occur, segmenting the data into distinct intervals. For each segment, the annual percent change (APC) and its 95% confidence interval (CI) were calculated to quantify the direction and magnitude of the trend.

Subgroup analyses were conducted according to sex and age, with age stratified into four brackets: 40–49, 50–59, 60–69, and ≥70 years. All age-standardized incidence rates (SIRs) were calculated using the 2019 Korean population structure as the reference to ensure comparability across years.

To visualize longitudinal changes in severe VI and the VI-to-incidence ratio, we applied cubic spline smoothing to annual incidence estimates from 2004 to 2019. The spline model facilitated graphical interpretation of gradual changes over time. Statistical analyses were performed using R (version 4.3.0, R Foundation for Statistical Computing, Vienna, Austria). Joinpoint regression analysis was conducted using Joinpoint Regression Program, Version 5.2.0, from the National Cancer Institute (NCI). Unless otherwise stated, data are presented as means ± standard deviations, and all tests were two-sided, with a significance threshold of *p* < 0.05.

### 2.6. Data Availability

The outflow of all data to the outside is strictly prohibited by national security law. The raw data used in this study can be extracted upon request from any qualified investigator through the National Health Insurance System service.

### 2.7. Ethics Statement

This study was approved by the Institutional Review Board of Seoul National University Hospital, Seoul, Korea (IRB no. 2306-006-1436). The requirement for informed consent was waived due to the use of anonymized and de-identified data. This study was conducted in accordance with the Declaration of Helsinki and complied with the STROBE reporting guidelines. Conditional access to the database was granted by the KNHIS Deliberative Committee.

## 3. Results

### 3.1. Overall Trends in Glaucoma Incidence

**Figure 1:**
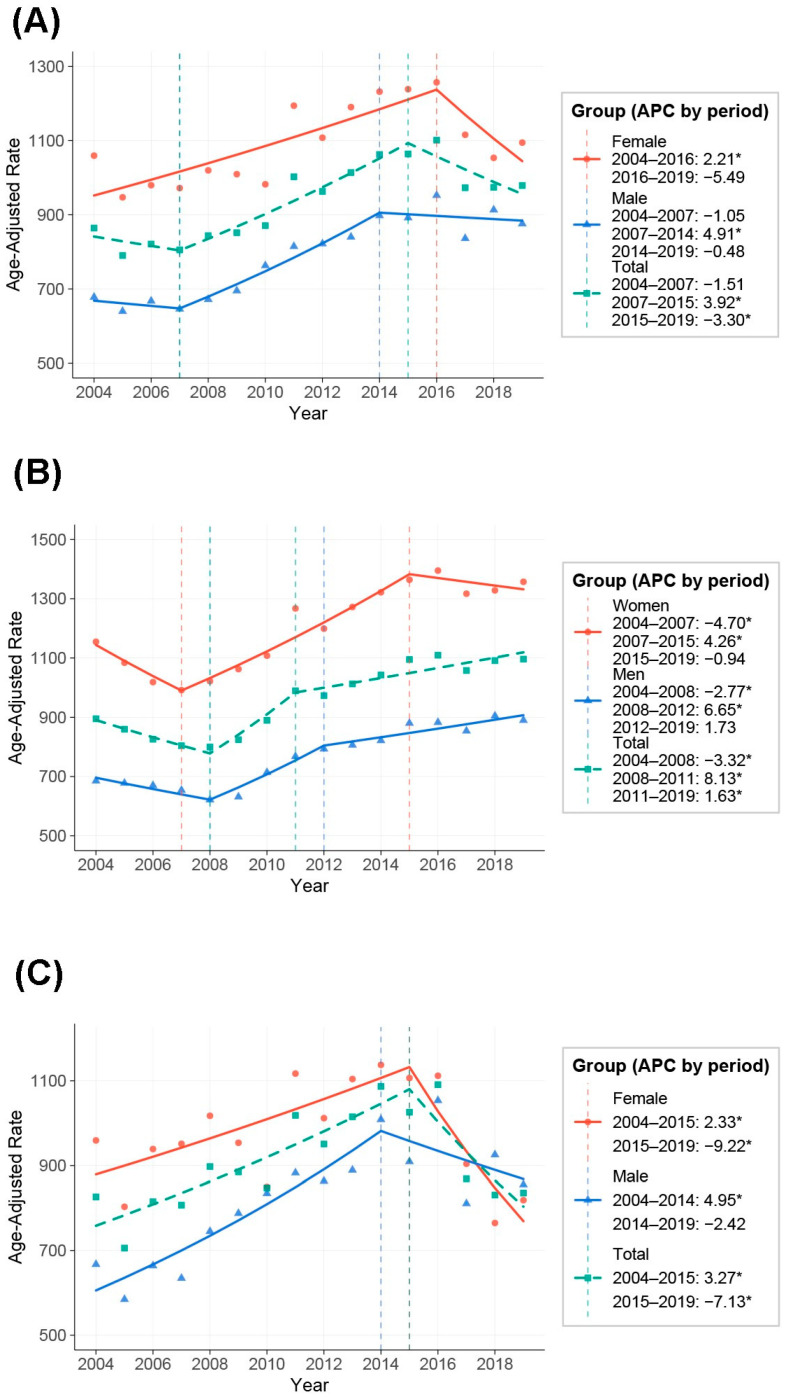
Joinpoint regression analysis of age-SIR of glaucoma (2004–2019). Trends in age-standardized incidence rates of glaucoma with identified joinpoints for (**A**) the total population, (**B**) individuals younger than 60 years, and (**C**) individuals aged 60 years and older. Annual percent changes (APCs) and their corresponding time intervals are indicated for each subgroup. Green indicates the total population, blue indicates males, and red indicates females. * statistically significant, *p* < 0.05.

The age-SIR of glaucoma was 864.1 per 100,000 individuals in 2004 and remained relatively steady until 2007, reaching 805.4 per 100,000. Thereafter, the age-SIR increased steadily to a peak of 1101.1 per 100,000 in 2016 (Table 1). However, after 2016, the SIR began to decline, reaching 978.9 per 100,000 in 2019. This biphasic pattern was confirmed in joinpoint regression, which identified a statistically significant increase from 2007 to 2015 (APC: +3.92%; 95% CI: 2.90 to 8.28; *p* = 0.003), followed by a decline from 2015 to 2019 (APC: –3.30%; 95% CI: –7.67 to –0.61; *p* = 0.018) (Figure 1A; Table S1).

The SIR remained consistently higher in females than in males throughout the study period (Table 1). The female-to-male (F–M) incidence ratio ranged from 1.15 to 1.56 during the study period. Specifically, in the 40–49, 50–59, and 60–69 age groups, the mean F–M ratios were 1.53 (95% CI: 1.50 to 1.57), 1.59 (95% CI: 1.56 to 1.63), and 1.47 (95% CI: 1.44 to 1.51), respectively, all statistically significant (*p* < 0.001). In contrast, among those aged ≥70 years, the F–M ratio was 1.02 (95% CI: 1.00 to 1.04; *p* = 0.021), indicating minimal sex differences in the older population (Table 2).

Among males, the incidence remained stable from 2004 to 2007, followed by a significant increase from 2007 to 2014 (APC: +4.91%; 95% CI: 3.42 to 9.73; *p* = 0.020), with a subsequent plateau. In contrast, females exhibited a significant upward trend from 2004 to 2016 (APC: +2.21%; 95% CI: 1.34 to 6.06; *p* = 0.006), followed by a decline with borderline significance (APC: –5.49%; 95% CI: –16.12 to 0.37; *p* = 0.077) (Table S1; Figure 1A).

### 3.2. Age-Specific Trends

Joinpoint regression analysis delineated age-specific trends. In the group aged <60 years, a transient decline was observed from 2004 to 2008 (APC: –3.32%; 95% CI: –8.49 to –1.19; *p* = 0.005), followed by a sharp increase from 2008 to 2011 (APC: +8.13%; 95% CI: 4.59 to 10.53; *p* < 0.001), and a continued but slower rise thereafter (2011–2019; APC: +1.63%; 95% CI: 0.35 to 2.26; *p* = 0.030). Among individuals aged ≥60 years, a consistent increase in incidence was noted from 2004 to 2015 (APC: +3.27%; 95% CI: 2.02 to 5.59; *p* < 0.001), which was followed by a significant decline from 2015 to 2019 (APC: –7.13%; 95% CI: –17.00 to –2.13; *p* = 0.008) (Figure 1B,C; Table S1).

### 3.3. Trends in Glaucoma-Related VI

The incidence rate of severe VI due to glaucoma declined markedly from 26.23 per 100,000 person-years in 2004 to 8.76 in 2012, and remained relatively stable thereafter, reaching 12.49 per 100,000 by 2019. From 2004 to 2012, the VI-to-incidence ratio decreased substantially, dropping from 0.030 to 0.009. This ratio subsequently plateaued and was 0.013 by the end of the study period in 2019 (Table 3). Joinpoint regression revealed that the severe VI incidence rate significantly declined from 2004 to 2012 (APC: –13.1%; 95% CI: –22.04 to –3.16; *p* = 0.016), followed by a non-significant change from 2012 to 2019 (APC: +4.7%; 95% CI: –8.34 to 19.54; *p* = 0.465) (Table S2). Throughout the study duration, females consistently showed lower standardized VI rates compared with males.

**Figure 2:**
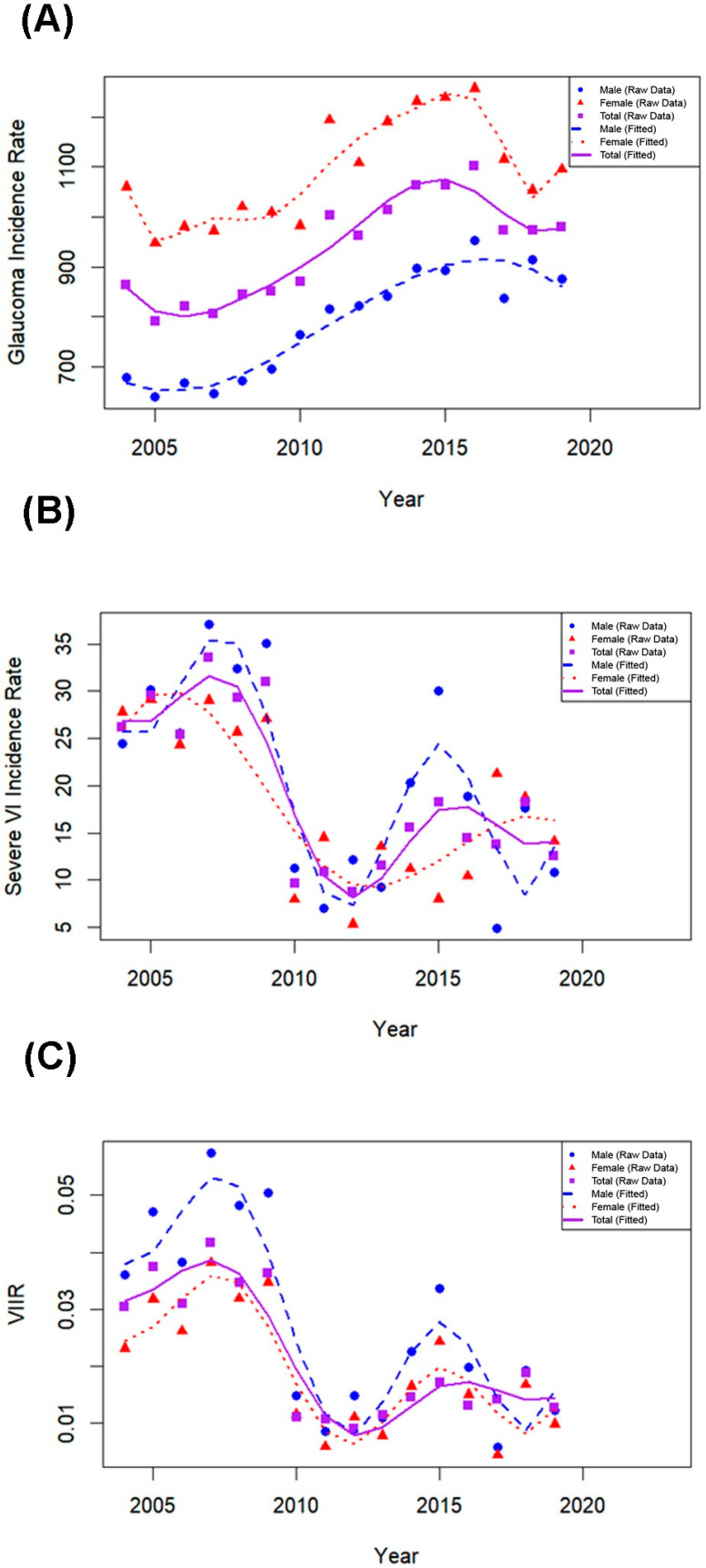
Trends in glaucoma incidence, severe VI, and their ratio by gender (2004–2019) using spline models. (**A**) Annual incidence rates of glaucoma (per 100,000 person-years) by gender. (**B**) Annual incidence rates of severe VI attributable to glaucoma by gender. (**C**) Annual ratio of VI to glaucoma incidence rates (VIIR) by gender. Dots indicate observed values; dotted and smoothed lines represent spline model fits. Purple indicates the total population, blue indicates males, and red indicates females.

Figure 2 presents the spline-smoothed trends of annual glaucoma incidence, severe VI incidence, and the corresponding VI-to-incidence ratios by gender from 2004 to 2019. A marked decline in both VI incidence and the relative risk of VI among glaucoma patients was observed until 2012, followed by a period of relative stabilization. Notably, males consistently exhibited higher rates of VI and a greater VI-to-incidence ratio compared with females throughout the study period.

## 4. Discussion

In this nationwide population-based analysis, we evaluated the longitudinal trends in the incidence of glaucoma and glaucoma-related severe VI in South Korea over a 16-year period. Our results demonstrated a biphasic pattern: a significant increase in glaucoma incidence between 2004 and 2015, which peaked around 2015, after which it plateaued and slightly declined. Second, and importantly, the incidence of severe VI attributable to glaucoma declined significantly until 2012, followed by a period of relative stabilization.

Our findings are consistent with previously reported trends in glaucoma incidence and offer additional insights. A previous nationwide analysis conducted between 2002 and 2013 reported that the glaucoma incidence in Korea approximately doubled (from 0.06% to 0.11% per year) over that period [4], reflecting the impact of heightened detection and an aging population. Our study builds upon this by demonstrating that the rise in incidence began to plateau around 2015. A similar plateauing trend has also been observed in a population-based study from Norway, a country with a comparably aging population [10].

Several plausible explanations can be proposed for the observed trends in glaucoma incidence. The steady rise in incidence through 2015 can be attributed to three main factors: population aging, advances in diagnostic modalities, and increased public awareness. South Korea’s population has been aging rapidly, and increased life expectancy enlarges the pool of older individuals at risk for glaucoma [4]. Concurrently, the widespread clinical adoption of OCT, from the late 2000s and the early 2010s [11], enabled earlier and more accurate detection of glaucoma that previously might have gone undiagnosed [5]. Over the past two decades, ophthalmologic societies and public health initiatives in Korea have actively promoted awareness of glaucoma, encouraging regular eye examinations and earlier referral for suspect cases [4]. Notably, the Korean Glaucoma Society (KGS) has actively participated in the annual World Glaucoma Week campaign, organized globally by the World Glaucoma Association (WGA) and the World Glaucoma Patient Association (WGPA) since 2008. This initiative has expanded in scope since 2010, with the KGS launching week-long awareness campaigns every year, which aimed at educating the public on the risks of glaucoma and the importance of regular ophthalmologic examinations for early detection of glaucoma. However, in our study, glaucoma was defined based on receipt of medical or surgical treatment, which does not include patients diagnosed with early-stage or preperimetric glaucoma who were not yet treated. It is plausible that, by around 2015, increased screening and awareness had saturated the pool of prevalent cases that would meet our treatment-based case definition. Consequently, the number of newly diagnosed and treated cases began to stabilize, leading to the observed plateau in incidence. Similar saturation-related plateaus have been observed in other chronic diseases, such as type 2 diabetes, in high-income settings [12].

The downward trend in glaucoma-related VI observed in our study is strongly supported by previous reports. Globally, the rate of blindness attributable to glaucoma appears to be decreasing in relative terms. From 1990 to 2020, the age-standardized prevalence of blindness and vision loss due to glaucoma decreased by 5.25 per 100,000 population, representing a 27.0% reduction [6], indicating a modest improvement despite the growing number of glaucoma cases. A long-term cohort study from Minnesota, US, also reported that the 10-year incidence of blindness in patients with open-angle glaucoma declined significantly, from 8.7 to 5.5 per 100,000, between cohorts diagnosed in 1965–1980 and 1981–2000, respectively (*p* = 0.02) [13]. Consistent with these global and regional findings, our results show a pronounced reduction in glaucoma-related VI incidence over time. In Korea, there has been little prior research quantifying trends in glaucoma-induced visual disability at the national level, so our finding of declining glaucoma-related VI risk is a particularly encouraging indication that modern glaucoma management is yielding tangible benefits in preserving vision.

The significant decrease in glaucoma-related VI incidence reflects improvements in clinical care and therapy. Treatment for glaucoma has advanced with the introduction of more effective intraocular pressure-lowering medications and the refinement of surgical and laser interventions. Such therapeutic advances, along with improved diagnostic monitoring (e.g., widespread use of OCT and visual field testing for the early detection of progression), have benefited patients [6,14,15]. We can speculate that glaucoma progression is now being detected more readily and at earlier stages, and that patients are also receiving better treatment, resulting in fewer individuals progressing to severe vision loss.

The gender disparity in glaucoma incidence has been controversial among various population-based studies [16,17,18,19,20,21]. Our study consistently showed that females had higher glaucoma incidence throughout the 16-year period, with F–M ratios ranging from 1.15 to 1.56, regardless of age group. Interestingly, our study showed a higher predisposition to glaucoma-associated VI among males. This result is consistent with previous studies regarding VI. Zeng et al. reported that the VI burden was higher in males than in females [22]. This disparity may be due to a combination of factors, including better adherence [23], higher health literacy [24,25], and more frequent utilization of screening or follow-up examinations among females [26]. Also, female sex hormones may exert a protective effect on glaucoma. A previous study demonstrated an increase in intraocular pressure following menopause [27]. Furthermore, the initiation of hormone replacement therapy has been associated with significant intraocular pressure reduction. The neuroprotective role of female sex hormones has also been supported by animal studies, which showed that 17β estradiol prevented retinal ganglion cell loss in a rat model of acute intraocular pressure elevation [28,29].

Despite the strengths of utilizing a comprehensive nationwide dataset, this study has several limitations, particularly those inherent to using claims-based data. First, the identification of glaucoma cases relied on diagnostic codes from insurance claims, meaning that only individuals who sought medical care and received a clinical diagnosis were included. To reduce potential misclassification, we restricted our analysis to patients with both a glaucoma diagnosis code and corresponding records of glaucoma medication use or surgical procedures. Meanwhile, the strict definition of glaucoma used in this study may have excluded individuals with preperimetric or asymptomatic glaucoma who had not yet initiated medication. This could have led to an underestimation of the glaucoma incidence and warrants caution when interpreting our findings. Second, changes in healthcare-seeking behavior, diagnostic criteria, or coding practices over time may have introduced classification bias. Third, the true burden of mild or asymptomatic glaucoma may have been underestimated, as individuals without healthcare utilization would not have been captured. Fourth, the claims data lacked clinical parameters such as intraocular pressure measurements, visual field indices, or treatment adherence, limiting our ability to assess disease severity or management outcomes. In addition, the limited clinical detail in the dataset made it impossible to distinguish between glaucoma subtypes such as normal-tension glaucoma and high-tension open-angle glaucoma. Plus, the role of recent advances in glaucoma treatment, such as novel medications and surgical techniques, could not be thoroughly assessed due to the lack of clinical detail in claims data. Further studies using clinical datasets are needed to explore their impact on visual outcomes. Fifth, the study period was limited to data up to 2019 due to access restrictions of the KNHIS database at the time of analysis. As such, the impact of more recent healthcare policies or epidemiologic changes could not be captured. Future studies incorporating updated datasets will be valuable in validating and expanding upon these findings. Lastly, temporal changes in diagnostic practices or thresholds over the 16-year study period may also have influenced the observed trends.

In conclusion, our findings demonstrate a significant increase in the incidence of glaucoma in Korea between 2004 and 2015, followed by a stabilization thereafter. Notably, the incidence of glaucoma-associated VI showed a plateauing trend after 2012. This pattern reflects the combined effects of public awareness, diagnostic advances, policy changes, and therapeutic improvements. Continued surveillance and targeted screening programs will be essential to reduce glaucoma-related VI in the aging Korean population.

## Figures and Tables

**Table 1:** Annual number of glaucoma cases and incidence rates (crude and age-standardized) by sex in Korea in 2004–2019 *.

		Number of Cases			Incidence Rate (Per 100,000 Person-Year)			
	Year	Total	Male	Female	Total	Male	Female	F–M Ratio
Crude	2004	4462	1860	2602	887.8	681.2	1133.8	1.66
	2005	4139	1782	2357	837.5	663.6	1044.5	1.57
	2006	4023	1756	2267	825.9	663.5	1019.1	1.54
	2007	3894	1681	2213	811.5	644.9	1009.6	1.57
	2008	3972	1654	2318	838.3	642.8	1070.8	1.67
	2009	3865	1634	2231	831.4	647.2	1050.2	1.62
	2010	4028	1795	2233	881.2	723.4	1068.5	1.48
	2011	4444	1858	2586	990.1	762.6	1260.2	1.65
	2012	4249	1868	2381	962.6	779.4	1180.3	1.51
	2013	4357	1866	2491	1010.1	796.9	1263.2	1.59
	2014	4462	1904	2558	1056.5	830.4	1325.1	1.60
	2015	4449	1959	2490	1076.3	872.4	1318.7	1.51
	2016	4425	1922	2503	1092.3	873	1353.4	1.55
	2017	4034	1756	2278	1022.3	818.5	1265.2	1.55
	2018	4005	1847	2158	1039.9	881.6	1228.6	1.39
	2019	3937	1765	2172	1047.7	863.5	1267.3	1.47
Standardized	2004	269,940	97,483	172,457	864.1	678	1059.4	1.56
	2005	246,162	91,990	154,172	790.3	639.8	947.1	1.48
	2006	255,472	95,983	159,489	821.2	667.6	979.7	1.47
	2007	251,101	92,877	158,224	805.4	646	972	1.50
	2008	262,601	96,604	165,997	843.8	671.9	1019.7	1.52
	2009	264,275	99,947	164,328	851.6	695.1	1009.4	1.45
	2010	269,601	109,715	159,886	870.8	763.1	982.2	1.29
	2011	311,514	117,137	194,377	1002.5	814.7	1194	1.47
	2012	298,503	118,174	180,329	963	821.9	1107.7	1.35
	2013	314,547	120,792	193,755	1013.7	840.1	1190.2	1.42
	2014	329,628	129,081	200,547	1062.5	897.8	1231.9	1.37
	2015	329,866	128,251	201,615	1063.8	892	1238.5	1.39
	2016	341,539	136,928	204,612	1101.1	952.3	1256.9	1.32
	2017	301,859	120,247	181,612	972.9	836.3	1115.6	1.33
	2018	302,794	131,308	171,486	974.1	913.2	1053.4	1.15
	2019	304,093	125,915	178,178	978.9	875.7	1094.5	1.25

* Standardized using the 2019 Korean standard population.

**Table 2:** Average age-specific standardized incidence rates of glaucoma and female-to-male ratios in 2004–2019 *.

Age Group, yr	Number of Cases			Incidence Rate (Per 100,000 Person-Year)								
	Total	Male	Female	Total	95% CI	Male	95% CI	Female	95% CI	F–M Ratio	95% CI	*p*-Value
40–49	26,360	12,497	13,863	786.39	780.37–792.41	622.92	615.4–630.44	955.10	945.64–964.56	1.53	1.50–1.57	<0.005
50–59	22,892	9708	13,184	1174.42	1167.14–1181.7	907.04	898.02–916.06	1444.89	1433.44–1456.34	1.59	1.56–1.63	<0.005
60–69	15,302	5836	9466	1214.48	1205.69–1223.26	977.61	966.34–988.88	1441.12	1427.73–1454.51	1.47	1.44–1.51	<0.005
70+	2191	866	1325	668.11	662.36–673.86	659.30	649.96–668.64	673.39	666.09–680.69	1.02	1.00–1.04	0.022

* Standardized using the 2019 Korean standard population.

**Table 3:** Annual glaucoma incidence, severe visual impairment (VI) incidence, and VI-to-incidence ratio by sex (2004–2019).

	Glaucoma Incidence Rate (Per 100,000)			Severe VI Incidence Rate (Per 100,000)			VI-to-Incidence Ratio		
Year	Total	Male	Female	Total	Male	Female	Total	Male	Female
2004	864.1	678	1059.4	26.23	24.47	27.81	0.030	0.036	0.026
2005	790.3	639.8	947.1	29.59	30.12	29.08	0.037	0.047	0.031
2006	821.2	667.6	979.7	25.39	25.6	24.32	0.031	0.038	0.025
2007	805.4	646	972	33.6	37.06	29.04	0.042	0.057	0.03
2008	843.8	671.9	1019.7	29.28	32.45	25.66	0.035	0.048	0.025
2009	851.6	695.1	1009.4	31.01	35.04	27.05	0.036	0.05	0.027
2010	870.7	763.1	982.2	9.62	11.27	7.92	0.011	0.015	0.008
2011	1002.5	814.7	1194	10.82	7	14.46	0.011	0.009	0.012
2012	963	821.9	1107.7	8.76	12.2	5.28	0.009	0.015	0.005
2013	1013.7	840.1	1190.2	11.53	9.22	13.5	0.011	0.011	0.011
2014	1062.5	897.8	1231.9	15.51	20.3	11.14	0.015	0.023	0.009
2015	1063.8	892	1238.5	18.27	30.09	7.99	0.017	0.034	0.006
2016	1101.1	952.3	1256.9	14.39	18.88	10.37	0.013	0.02	0.008
2017	972.9	836.3	1115.6	13.75	4.92	21.25	0.014	0.006	0.019
2018	974.1	913.2	1053.4	18.26	17.6	18.72	0.019	0.019	0.018
2019	978.9	875.7	1094.5	12.49	10.78	14.09	0.013	0.012	0.013

## Data Availability

All data generated and analyzed during the current study are available at the National Health Insurance Data-Sharing Service (available online at https://nhiss.nhis.or.kr/bd/ab/bdaba000eng.do, accessed on 31 August 2024).

## Supplementary Materials

The following supporting information can be downloaded at https://www.mdpi.com/article/10.3390/jcm14165691/s1. Table S1: Joinpoint regression analysis of age-standardized glaucoma incidence rates in Korea (2004–2019); Table S2: Joinpoint regression analysis of severe VI incidence rates in Korea (2004–2019).

## Abbreviations

The following abbreviations were used in this manuscript:

APC	Annual percent change
CI	Confidence interval
KNHIS	Korean National Health Insurance Service
OCT	Optical coherence tomography
SIR	Standardized incidence rate
VI	Visual impairment
