# Supplementary material for: Recent Changes in Trends of Nationwide Incidence of Glaucoma and Associated Visual Impairment in South Korea
Source: J Clin Med. 2025 Aug 12;14(16):5691. doi: 10.3390/jcm14165691 (PMC12386786; doi:10.3390/jcm14165691)
Supplement: Supplementary file 1 [file jcm-14-05691-s001.zip › jcm-3777248-supplementary.pdf]

Supplementary Materials

Recent Changes in the Trends of Nationwide Incidence of Glaucoma and Associated Visual Impairment in South Korea

Table S1. Joinpoint Regression Analysis of Age-Standardized Glaucoma Incidence Rates in Korea (2004–2019)

Table S2. Joinpoint Regression Analysis of Severe VI Incidence Rates in Korea (2004–2019)

Table S1. Joinpoint Regression Analysis of Age-Standardized Glaucoma Incidence Rates in Korea (2004–2019).

Segment	Years	APC	95% CI	*p* Value	APC	95% CI	*p* Value	APC	95% CI	*p* Value
All age group		Total			Male			Female		
1st	2004–2007	-1.51	–6.83 to 2.58	0.49	-1.05	–6.78 to 3.50	0.628	2.21	1.34 to 6.06	0.006
2nd	2007–2015	3.92	2.90 to 8.28	0.003	4.91	3.42 to 9.73	0.02	-5.49	–16.12 to 0.37	0.077
3rd	2015–2019	-3.3	–7.67 to –0.61	0.018	-0.48	–4.26 to 1.46	0.603			
< 60										
1st	2004–2008	-3.32	–8.49 to –1.19	0.005	-2.77	–8.16 to –0.43	0.023	-4.7	–8.49 to –1.71	0.005
2nd	2008–2012	8.13	4.59 to 10.53	<0.001	6.65	4.28 to 10.22	<0.001	4.27	3.55 to 6.66	<0.001
3rd	2012–2019	1.63	0.35 to 2.26	0.03	1.73	–0.004 to 2.53	0.05	–0.94	–5.38 to 1.11	0.306
≥ 60										
1st	2004–2014	3.27	2.02 to 5.59	<0.001	4.95	3.54 to 8.43	0.0004	2.33	0.93 to 4.65	<0.001
2nd	2014–2019	-7.13	–17.00 to –2.13	0.008	-2.42	–10.33 to 1.05	0.171	-9.22	–19.68 to –3.81	<0.001

Table S2. Joinpoint Regression Analysis of Severe VI Incidence Rates in Korea (2004–2019)

Groups	Years	APC	95% CI	*p* value
Total				
1st	2004–2012	-13.1106*	[-22.04, -3.16]	0.016
2nd	2012–2019	4.6725	[-8.34, 19.54]	0.465
Male				
1st	2004–2011	-13.0158	[-30.14, 8.31]	0.189
2nd	2011–2019	-1.0330	[-17.25, 18.37]	0.901
Female				
1st	2004–2012	-16.3224	[-53.27, 49.33]	0.067
2nd	2012–2019	9.3746	[-35.21, 100.92]	0.230
